# Dengue virus introduction and 2025 outlook: scenario-based evaluation of vaccination impact using mathematical modeling

**DOI:** 10.1590/0102-311XEN150325

**Published:** 2026-06-26

**Authors:** Thomas Nogueira Vilches, Cláudia Pio Ferreira, Miguel Rubira Telles, Lais Moraes Paiz, Gabriel Berg de Almeida

**Affiliations:** 1 Instituto de Biociências de Botucatu, Universidade Estadual Paulista “Júlio de Mesquita Filho”, Botucatu, Brasil.; 2 Faculdade de Medicina de Botucatu, Universidade Estadual Paulista “Júlio de Mesquita Filho”, Botucatu, Brasil.

**Keywords:** Basic Reproductive Number, Immunization Programs, Epidemiological Models, Número Reprodutivo Básico, Programas de Imunização, Modelos Epidemiológicos, Número Reproductivo Básico, Programas de Inmunización, Modelos Epidemiológicos

## Abstract

This study aims to model multiple scenarios of dengue introduction and spread in Botucatu, São Paulo State, Brazil, and to evaluate the impact of vaccination strategies on the epidemic trajectory in a city that, until 2023, had less than 1% of its population infected with dengue. First, we estimated the basic reproduction number (*R*_*0*_ ) during the 2024 epidemic and compared it with values reported in the literature. We then developed an age-stratified mathematical model, calibrated to 2024 dengue case data using a genetic algorithm, to simulate transmission dynamics with and without vaccination. This approach enabled us to assess the potential reduction in infections under various immunization scenarios. The estimated *R*_*0*_ for the 2024 epidemic was 1.57, resulting in an attack rate exceeding 10% of the population. Our model accurately fits the observed data and suggests that, under conditions similar to those of 2024, the introduction of a new serotype in 2025 would likely trigger another epidemic. Vaccination could reduce this peak by up to 80%, depending on coverage among individuals aged 10-14 years. The results indicate that the *R*_*0*_ of dengue estimated for Botucatu in 2024 is consistent with values reported for other Brazilian cities. The vaccination campaign shows the potential to reduce dengue cases by 75% or more. However, since campaign effectiveness depends on the circulating serotype and the epidemiological status of vaccinated individuals, achieving vaccination coverage above 50% in the target population is essential to avoid the need for individual epidemiological screening.

## Abstract (pt)

O objetivo deste estudo foi modelar múltiplos cenários de introdução e disseminação da dengue em Botucatu, São Paulo, Brasil, e avaliar o impacto das estratégias de vacinação na trajetória epidêmica de uma cidade que tinha menos de 1% de sua população infectada com dengue até 2023. Estimamos o número básico de reprodução (*R*_*0*_ ) durante a epidemia de 2024 e o comparamos com os valores relatados na literatura. Em seguida, desenvolvemos um modelo matemático estratificado por idade (calibrado para dados de casos de dengue de 2024 por um algoritmo genético) para simular a dinâmica de transmissão com e sem vacinação. Isso nos permitiu avaliar a possível redução de infecções em vários cenários de vacinação. O *R*_*0*_ estimado para a epidemia de 2024 foi de 1,57, levando a uma taxa de ataque superior a 10% da população. Nosso modelo se ajusta com precisão aos dados observados e sugeriu que, sob as mesmas condições de 2024, a introdução de um novo sorotipo em 2025 provavelmente desencadearia outra epidemia. A vacinação pode reduzir esse pico em até 80%, dependendo da cobertura entre indivíduos de 10-14 anos. Os resultados indicam que o *R*_*0*_ da dengue em Botucatu em 2024 é consistente com estimativas para outras cidades do Brasil. A campanha de vacinação pode reduzir os casos de dengue em 75% ou mais. No entanto, como a eficácia da campanha depende do sorotipo circulante e do *status* epidemiológico dos indivíduos vacinados, alcançar cobertura vacinal acima de 50% na população-alvo é essencial para evitar a necessidade de triagem epidemiológica individual.

## Abstract (es)

El objetivo de este estudio es modelar múltiples escenarios de introducción y propagación del dengue en Botucatu, São Paulo, Brasil, y evaluar el impacto de las estrategias de vacunación en la trayectoria epidémica en una ciudad que, hasta 2023, tenía menos del 1% de su población infectada por el dengue. En primer lugar, estimamos el número básico de reproducción (*R*_*0*_ ) durante la epidemia de 2024 y lo comparamos con los valores reportados en la literatura. A continuación, desarrollamos un modelo matemático estratificado por edades, calibrado con los datos de casos de dengue de 2024 utilizando un algoritmo genético, para simular la dinámica de transmisión tanto con como sin vacunación. Esto nos permitió evaluar la posible reducción de las infecciones en diversos escenarios de vacunación. La estimación de *R*_*0*_ para la epidemia de 2024 fue de 1,57, lo que daría lugar a una tasa de ataque superior al 10% de la población. Nuestro modelo se ajusta con precisión a los datos observados y sugiere que, en las mismas condiciones que en 2024, la introducción de un nuevo serotipo en 2025 probablemente desencadenaría otra ola epidémica. La vacunación podría reducir este pico hasta en un 80%, dependiendo de la cobertura entre las personas de 10-14 años. Los resultados indican que el *R*_*0*_ la epidemia de dengue en Botucatu, para 2024, es coherente con las estimaciones de otras ciudades de Brasil. La campaña de vacunación muestra el potencial de reducir los casos de dengue en al menos un 75%. Sin embargo, dado que la eficacia de la campaña depende del serotipo circulante y del estado epidemiológico de las personas vacunadas, es esencial lograr una cobertura de vacunación superior al 50% en la población objetivo para evitar la necesidad de realizar pruebas epidemiológicas individuales.

## Introduction

Dengue is the most significant arboviral disease worldwide, with the highest estimated global prevalence and a substantial impact on public health ^1^. It is caused by a virus of the *Flavivirus* genus and is transmitted primarily by *Aedes aegypti* and *Aedes albopictus* mosquitoes ^2^. The disease affects tens of millions of people yearly, with approximately 16.2 million cases reported in the Americas alone, contributing to recurrent epidemics and placing a considerable burden on health systems ^3^. Clinical manifestations range from asymptomatic infection to severe and potentially fatal forms ^4^. Infection may be caused by four distinct virus serotypes (DENV-1 to DENV-4), and recovered individuals acquire permanent immunity to the homologous serotype and temporary immunity to heterologous serotypes, such that reinfections are observed ^5^.

Tropical and subtropical regions are disproportionately affected, particularly the Caribbean, South America, and Southeast Asia, where high endemicity has been documented ^6^. In addition, dengue has expanded in both geographical range and incidence. Reports indicate an increase in cases from 500,000 in 2000 to 2.4 million in 2010 and 5.2 million in 2019 ^7^. Countries that historically did not report autochthonous dengue cases, such as France, Italy, Spain, Croatia, and even the United States, are now documenting substantial transmission, further increasing the global burden of the disease ^8^.

Multiple factors contribute to the spread of dengue. Climate change, characterized by rising global temperatures and extreme weather events such as intense rainfall and heat waves, facilitates the expansion of the vector. Furthermore, unplanned urban growth, inadequate waste management, poor sanitation infrastructure, and ineffective public vector control measures exacerbate this scenario ^9^ ^,^ ^10^. Together, these factors contribute not only to the active dispersion of the vector but also to the spread of multiple dengue virus serotypes.

Brazil exemplifies this concerning trend, with widespread distribution of *Ae. aegypti* across its territory and the circulation of all four dengue virus serotypes. According to the Brazilian Ministry of Health, more than six million probable dengue cases were recorded in 2024, accounting for 67.4% of all cases in Latin America and the Caribbean ^11^. Major epidemics are often associated with the introduction of a new serotype or the reemergence of a serotype that has been absent for an extended period ^12^.

From 2000 to 2024, Brazil reported more than 23 million cases of dengue ^13^, reflecting its persistent endemic status. However, Brazil’s large dimensions and regional disparities create a diverse epidemiological landscape. Dengue has progressively expanded into regions previously considered low risk, such as southern states, which historically exhibited milder climates and environmental conditions unfavorable to *Aedes* proliferation ^14^. Brazil ended 2022 with a record of 1,016 deaths caused by dengue, as reported by the Brazilian Ministry of Health Epidemiological Bulletin ^15^. This is the highest number of dengue-related deaths ever recorded since surveillance began ^16^.

A particularly illustrative case is the municipality of Botucatu, located in the south-central region of São Paulo State, Brazil, approximately 235km from the state capital. With a population of just over 150,000 inhabitants, the municipality comprises well-defined urban and rural areas surrounded by native *cerrado* and Atlantic forest vegetation. Historically, high elevation, low humidity, native vegetation, and temperate climate acted as ecological barriers to *Ae. aegypti* migration. Rugged topography and lower population density further hindered the establishment of breeding sites, contributing to low transmission rates for decades. However, over the past 10 years, accelerated urbanization, unregulated growth in peripheral neighborhoods, and rising summer temperatures and humidity have disrupted this balance. Consequently, Botucatu transitioned from a low-risk area to one experiencing sustained and recurrent dengue transmission.

Prior to 2015, Botucatu had no significant record of autochthonous dengue transmission. However, in that year, the city reported 879 locally acquired cases ^13^. In subsequent years, the number of confirmed cases remained relatively low, reaching a cumulative total of approximately 1,800 by 2023. In 2024, the municipality experienced an unprecedented outbreak, with 17,007 confirmed cases and 14 deaths. The predominant serotype identified was DENV-1, suggesting the initial introduction of the virus into a largely susceptible population. The outbreak had a profound impact on the city, necessitating urgent public health interventions. A dengue emergency was declared by municipal decree ^17^. These measures included the establishment of emergency hydration centers, extended shifts in primary care units, increased hospital bed offer, and intensified vector control actions, such as insecticide fogging in strategic areas ^18^.

Historically, dengue control has relied on vector control strategies, whose efficacy has become increasingly limited. Environmental and behavioral changes such as climate shifts, inadequate urban infrastructure, poor sanitation, and vector adaptation have compromised traditional approaches to disease control. *Ae. aegypti*, once a peridomestic vector, has evolved into a highly urbanized, indoor-dwelling mosquito that endures conventional interventions ^9^. In this context, novel preventive strategies are urgently needed. Although dengue vaccine development has been ongoing for years, few candidates have demonstrated adequate safety profiles for public health use ^19^. Qdenga (TAK-003), a live-attenuated tetravalent vaccine based on a DENV-2 backbone with structural genes from the other three serotypes, emerged as a promising alternative. Phase 3 trials demonstrated approximately 62% efficacy against symptomatic dengue and over 80% efficacy against hospitalization; however efficacy varied among the four serotypes ^20^.

In 2024, the Brazilian Ministry of Health incorporated Qdenga into the National Immunization Program (PNI, acronym in Portuguese). Given global demand and limited supply, initial vaccination efforts targeted hyperendemic areas, focusing on children and adolescents aged 10-14 years, using a two-dose schedule administered three months apart. Due to its previously low cumulative incidence, Botucatu was not included in this first phase of vaccination. This unique scenario makes Botucatu an ideal setting for a mathematical modeling study of dengue introduction in an almost fully susceptible population. It enables the estimation of key transmission parameters, such as the basic reproduction number. Thus, critical questions arise: What can be expected in 2025 with continued circulation of the same serotype under conditions similar to those of 2024? What are the possible outcomes of the introduction of a new serotype? How would partial vaccination coverage influence transmission dynamics?

## Methods

To conduct this epidemiological modelling study, secondary data from individual notifications of suspected dengue fever cases were used, retrieved from the Brazilian Information System for Notifiable Diseases (SINAN, cronym in Porgtuguese) of the municipality of Botucatu ^13^. The data are public and anonymized, and each row of the dataset corresponds to a notified dengue case (confirmed or discarded) ^21^. Data were filtered to include only confirmed cases for the municipality of Botucatu. Subsequently, the data were aggregated by Epidemiological Week of notification and age group. Age distribution in Botucatu was obtained from data provided by the Brazilian Institute of Geography and Statistics (IBGE, acronym in Portuguese) ^22^. The study included confirmed dengue fever cases among residents of Botucatu that occurred during the first epidemiological weeks of 2024.

### *R*_*0*_ estimation

According to Pinho et al. ^23^, *R*_*0*_ can be estimated using the following expression:

$$R_{0}=\sqrt{\left(1+\frac{\Lambda }{\mu_{m}}\right)\left(1+\frac{\Lambda }{\gamma }\right)\left(1+\frac{\Lambda }{\eta }\right)\left(1+\frac{\Lambda }{\nu_{m}+\mu_{m}}\right)}$$ (1)

The parameter *µ*_*m*_ denotes the mosquito mortality rate (1/3 week^-1^) ^23^ ^,^ ^24^; *γ* is the inverse of the infectious period in humans (1.0 week^-1^) ^24^; and *η* and *ν*_*m*_ represent the inverses of the latent periods in humans and mosquitoes, respectively (1.4 and 1.0 week^-1^) ^25^. *Λ* corresponds to the exponential growth rate at the onset of the epidemic and was estimated by fitting a linear regression to cumulative versus new dengue case data during the initial outbreak phase.

### Mathematical model

The time-discrete model considers that four serotypes can circulate simultaneously, and the subscript *j*, ranging from 1 to 4, denotes the serotype. In total, three age groups were considered: 0-9 years, 10-14 years, and 15 years and older. The subscript *a* denotes the age group. This stratification was based on the dengue vaccination strategy adopted in Brazil. The variable *S*_*a*_ represents susceptible individuals in age group *a*; *E*_*j,a*_ denotes latent individuals with serotype *j* and age group *a*; *I*_*j,a*_ represents infectious individuals infected with serotype *j* in age group *a*; *R*_*j,a*_ denotes individuals recovered from serotype *j* in age group *a*; *S*_*j,a*_ represents individuals recovered from serotype *j* and age group *a* but susceptible to other serotypes; *E*_*j2,a*_ denotes the latent compartment for secondary infection with serotype *j* in age group *a*; *I*_*j2,a*_ represents infectious individuals with secondary infection by serotype *j* in age group *a*; and *R*_*a*_ denotes individuals recovered from secondary infections in age group *a*. The model assumes that reinfection is possible only once; therefore, after a secondary infection with a heterologous serotype, individuals are considered immune to all subsequent serotypes. In the model, *X* ^*t*^ represents the number of individuals in state X∈S,E,I,R at time t, and ∆t is the time step. Thus,

Sat+Δt=Sate-∑iλi(a,t)Δt,Ej,at+Δt=Sat(1-e-λj(a,t)Δt)+Ej,ate-ηΔt,Ij,at+Δt=Ej,at(1-e-ηΔt)+Ij,ate-γΔt,Rj,at+Δt=Ij,at(1-e-γΔt)+Rj,ate-ϵΔt,Sj,at+Δt=Rj,at(1-e-ϵΔt)+Sj,ate-ξΔt∑i,i≠jλi(a,t),Ej2,at+Δt=∑i,i≠jSi,at(1-e-ξλj(a,t)Δt)+Ej2,ate-ηΔt,Ij2,at+Δt=Ej2,at(1-e-ηΔt)+Ij2,ate-γΔt,Rat+Δt=∑iIi2,at(1-e-γΔt)+Rat,fori,j=1,2,3,4. (2)

The parameters are defined as follows: ϵ represents the rate of loss of cross-immunity, while γ and η are the inverses of the infectious and latent periods, respectively. In addition, ξ denotes the influence of primary infection on transmission during secondary heterologous infection; specifically, if ξ > 1, the force of infection increases, whereas if ξ < 1, it decreases. The special case ξ = 1 means that the secondary heterologous infection is not influenced by immunological memory from the first infection.

The force of infection for serotype j in a susceptible individual belonging to age group a, is given by:

$$\lambda_{j}\left(a,t\right)=\beta_{h}\times b_{a}\times V_{j}\times \tau \left(t\right)$$ (3)

In which β_*h*_ is the probability of virus transmission from vector to humans, b_*a*_ corresponds to the number of bites per unit of time that a mosquito performs on individuals in age group a, and τ accounts for seasonal variation in dengue transmission, given by:

$$\tau \left(t\right)=0.5+0.5sin\left(\frac{2\pi t}{52}+\delta \right)$$ (4)

Specifically, following the approach of Vilches et al. ^26^ and Camargo et al. ^27^, the compartment representing vectors infected with dengue serotype j is described by:

$$V_{j}=\frac{\beta_{v}\sum_{a}b_{a}\left(I_{j,a}+I_{j_{2},a}\right)}{\mu_{m}+\sum_{a}b_{a}\sum_{i}^{4}\left(I_{i,a}+I_{i_{2},a}\right)}$$ (5)

In this expression, I_*j,a*_ + I_*j2,a*_ represents the proportion of humans infected with serotype j, including both primary and secondary infections, in age group a. The parameter β_*v*_ denotes the probability of virus transmission from humans to vectors, and µ_*m*_ represents the mosquito mortality rate. The model does not account for movement between age groups or demographic processes (natural mortality, births, or migration), as the simulated period is short enough (less than 360 days) to justify neglecting these factors. However, these processes can be readily incorporated to generalize the model.

### Model parameterization

Before 2024, dengue circulation in Botucatu was negligible. Furthermore, serological tests indicate that a unique serotype was circulating in Botucatu during the 2024 epidemic; therefore, the model was fitted considering this serotype. Therefore, we were able to set arbitrary values for ϵ and ξ . For further simulations considering new serotypes in the municipality, we used ϵ = 1/26 week^-1^ and ξ = 0.5, 1.0, and 1.5 ^28^. The parameters β_*h*_ (DENV transmission probability from mosquito to human), β_*v*_ (DENV transmission probability from human to mosquito), b_*a*_ (number of bites on age group a per mosquito), δ (sinusoidal-function phase), and the number of initial infectious individuals, 𝐼 1,𝑎 0 , were estimated from data. For this purpose, we used a genetic algorithm (GA) that maximizes a score L, defined as the sum of the inverse squared error when comparing observed data and model simulations for each age group a ^29^, as follows:

$$L=\sum_{a}\frac{n_{a}}{\sum_{i}^{n_{a}}{\left(y_{a}^{i}-d_{a}^{i}\right)}^{2}}$$ (6)

In this expression, 𝑦 𝑎 𝑖 is the simulated number of cases in week i for age group a, 𝑑 𝑎 𝑖 is the reported number of cases in week i for the same age group, and n_*a*_ is the number of individuals in age group a.

The GA was run for 150 generations, evaluating 3,000 different parameter sets sampled from uniform distributions using a Latin hypercube sampling (LHS) process ^30^. The assumed LHS sampling intervals were: b_*a*_ from 0.5 to 15 bites per week; β_*h*_ from 0.0001 to 0.3; β_*v*_ from 0.01 to 0.011, this parameter is assumed to have low variability, as it has high dependency on β_*h*_ and b_*a*_ values; δ from 0 to π/2; and 𝐼 1,𝑎 0 from 1 to 100.

### Setting up vaccination scenarios

The model does not explicitly include vaccinated individuals. However, vaccination was represented by the recovered class (R). We assume that the vaccine, administered at coverage (ν_*v*_ ), confers immunity against all four serotypes with probability p_*e*_ , which reflects its effectiveness. The proportion p of individuals who acquire immunity is:

$$p=p_{e}\times \nu_{v}$$ (7)

To simulate vaccination scenarios, we modified the initial conditions by assigning different numbers of immune individuals - corresponding to various p values - within the 10-14-year-old age group when running the model.

### AI usage 

All results and discussions were generated, developed, and interpreted solely by the authors. Artificial intelligence (ChatGPT 4.0) was used to review the English language, orthography, and reading flow. 

## Results

**Figure 1:**
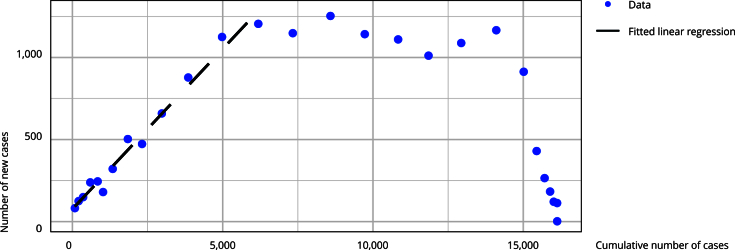
Weekly number of new dengue cases versus cumulative number of cases in Botucatu, São Paulo State, Brazil.

Note: the dashed line represents the linear regression fitted to data collected up to the 10^th^ Epidemiological Week of 2024. The estimated angular coefficient is Λ = 0.195 week^-1^.

Figure 1 shows the cumulative number of cases versus the number of new cases, as well as the linear regression obtained by fitting the first 10 weeks of data, which correspond to the exponential growth phase of the 2024 Botucatu epidemic curve. This enabled the estimation of the exponential growth rate, Λ = 0.195 week^-1^ (95% confidence interval - 95%CI: 0.177-0.213), and the basic reproduction number, R_*0*_ = 1.57 (95%CI: 1.52-1.63), using equation (1) and the parameter values given in the section R_*0*_ Estimation.

**Figure 2:**
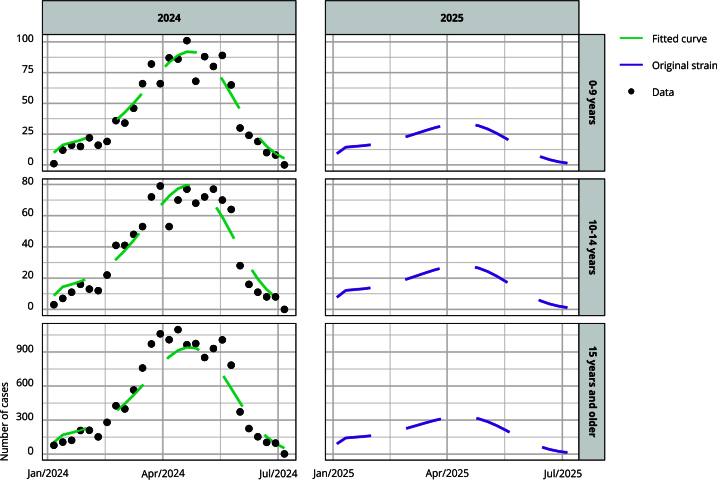
Incidence of dengue cases in 2024 and the counterfactual scenario of population immunity in 2025, considering the number of infections observed in the previous year and circulation of a homologous strain.

Note: reported data from 2024 are shown as black dots for the three age groups. The parameters estimated by the genetic algorithm (GA) are b_*a*_ = [11.5, 19.4, 17.1] week^-1^, for each age group; b_*h*_ = 0.054; b_*v*_ = 0.0102; d = 0.48; and 𝐼 1,𝑎 0 = [24, 43, 35] individuals in each age group respectively. The other fixed parameters are µ_*m*_ =1/3 week^-1^, γ = 1.0 week^-1^; and η = 1.4 week^-1^.

Figure 2 presents the dengue data stratified by age group, together with the corresponding curves fitted by the GA for 2024 (left panels). We used data from January to July to fit the model up to the epidemic peak (Epidemiological Weeks 1 to 27). The parameters estimated by the GA were b_*a*_ = [11.5, 19.4, 17.1] week^-1^ for each age group; b_*h*_ = 0.054; b_*v*_ = 0.0102; d = 0.48; and 𝐼 1,𝑎 0 = [24, 43, 35] individuals in each age group, respectively. Figure 2 also compares the fitted curve (green line) with a counterfactual scenario (purple line), in which the DENV-1 serotype re-emerges in 2025 under the same conditions as in 2024, but with population immunity shaped by the previous epidemic peak. From top to bottom, the panels show the results for the following age groups: 0-9, 10-14, and over 15 years. In the counterfactual scenario, the epidemic peak in the 10-14-year-old age group would reach approximately 37.5% of the size observed at the 2024 peak.

**Figure 3:**
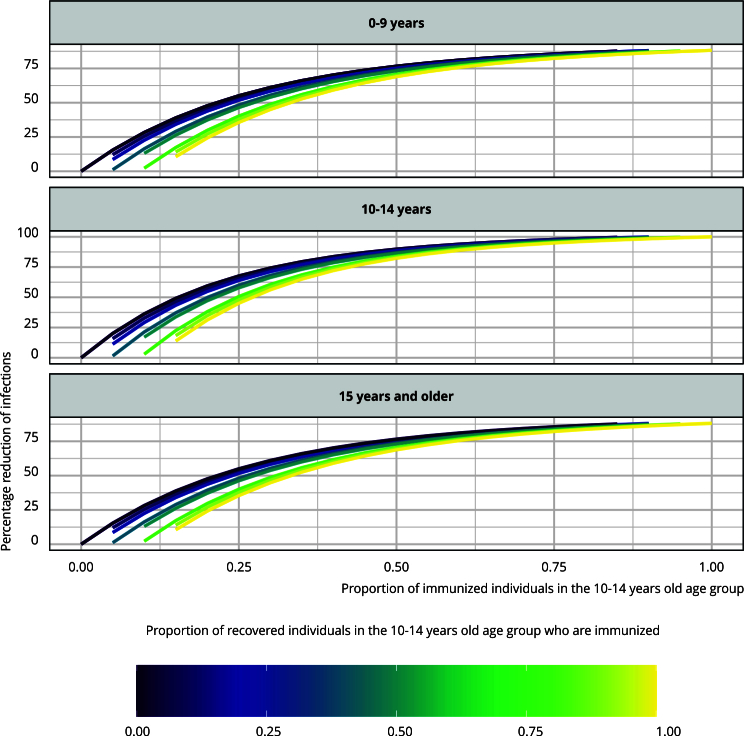
Reduction in the number of infections when comparing scenarios with and without vaccination across the three age groups.

Note: parameters are the same as in Figure 2. Initial conditions are modified to represent vaccine-induced immunity by transferring susceptible individuals to the recovered compartment.

To investigate how vaccination could reduce the number of infections in 2025 if it had been implemented in 2024, we ran simulations varying the proportion of immune individuals p, as presented in the previous section. We then quantified the reduction in the attack rate by comparing the vaccination scenarios with the no-vaccination scenario (baseline), represented by the purple curve in Figure 2. Figure 3 shows the results obtained. The effectiveness of the vaccination strategy depends on the immune status of the target group. The greater the proportion of vaccinated individuals who had previously recovered from DENV-1, the lower the net efficiency gained by the vaccination campaign.

Specifically, the data comprise 8,962 individuals aged 10 to 14 years, of whom 1,186 were infected during the 2024 epidemic peak. A vaccine-induced immunity of 25% of the population corresponds to 2,241 immune individuals. However, if all recovered individuals are included among those vaccinated, only approximately 1,055 new immune individuals would be generated through vaccination, resulting in a total of 2,241 individuals immune to DENV-1 (light-yellow line in Figure 3). In contrast, if priority is given to individuals who were not previously infected and no recovered individuals are vaccinated, the total number of individuals immune to DENV-1 reaches 3,427, leading to a greater reduction due to vaccination (dark-purple line in Figure 3).

**Figure 4:**
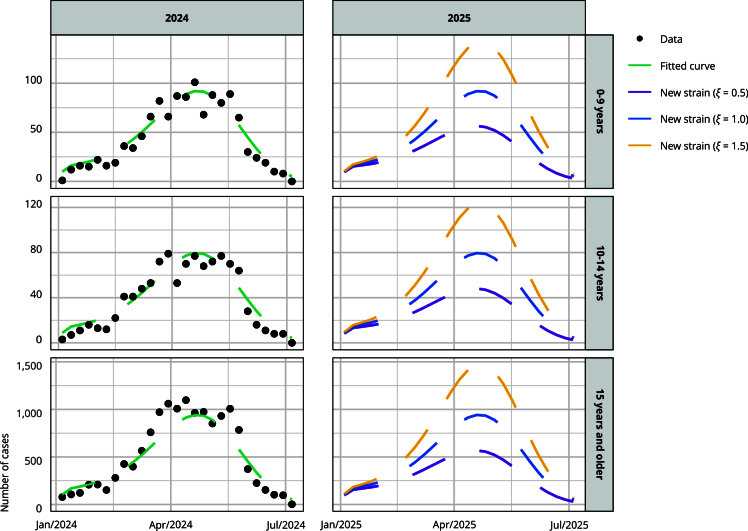
Incidence of dengue cases for the fitted curve and the counterfactual population immunity scenario in 2025, considering the number of infections in 2024 and the spread of a heterologous strain.

Note: parameters are b_*a*_ = [11.5, 19.4, 17.1] week^-1^, for each age group; b_*h*_ = 0.054; b_*v*_ = 0.0102; d = 0.48, µ_*m*_ = 1/3 week^-1^, γ = 1.0 week^-1^, η = 1.4 week^-1^ and ϵ = 1/26 week^-1^. The initial conditions for infected compartments are 𝐼 2,𝑎 0 = [24, 43, 35] individuals in each age group, respectively. Reported data from 2024 are also shown, and the panels correspond to the three age groups.

We also simulated a scenario in which a heterologous serotype enters the population in 2025 under the same conditions as in 2024. We considered three cases describing how primary infection influences secondary infection: susceptible individuals who have recovered from the primary infection are equally susceptible (ξ = 1.0), 50% less susceptible (ξ = 0.5), or 50% more susceptible (ξ = 1.5) than individuals without prior infection. As in Figure 3, the results were stratified by age group, and the data and fitted curve of the 2025 epidemic were retained for comparison. When ξ = 1.5, the epidemic peak increases because the entire population is susceptible to the new serotype, with a subset experiencing an increased infection rate. Conversely, when ξ = 0.5, the epidemic peak decreases. When ξ = 1.0, the epidemic peak under the same conditions as in 2024 is identical to the peak observed in 2024. These results are shown in Figure 4.

**Figure 5:**
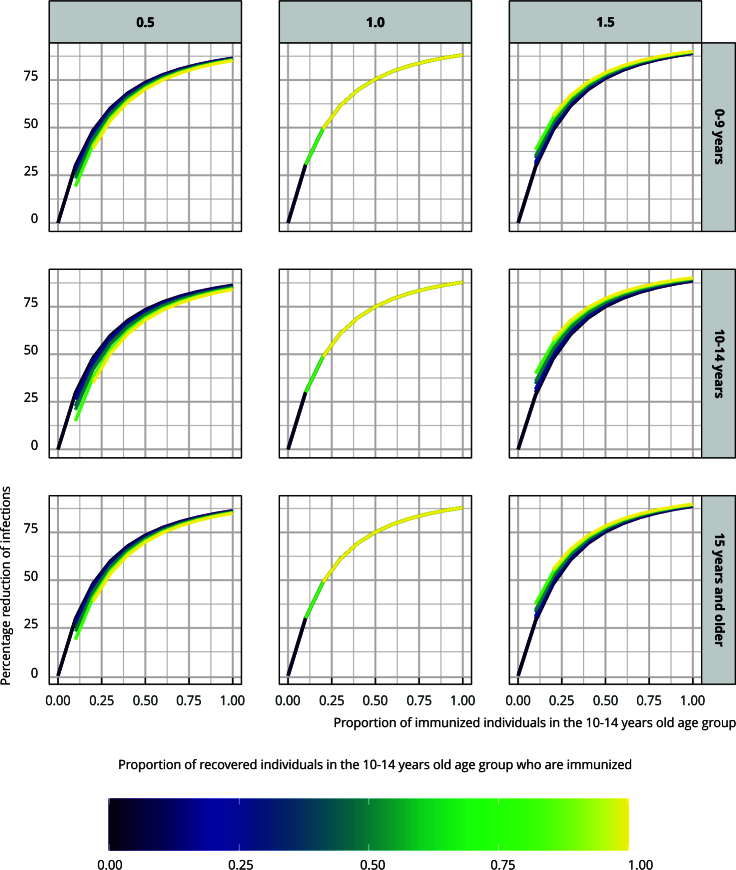
Reduction in the number of infections between scenarios with and without vaccination across the three age groups.

Note: parameters are the same as in Figure 4. Initial conditions are modified to represent vaccine-induced immunity by transferring susceptible individuals to the recovered compartment. Each column of panels represents a different value of ξ.

Using the same scenarios from Figure 4 as baselines (represented by the orange, blue, and purple curves), we simulated different vaccination coverages - analogous to those in Figure 3 - to estimate the reduction in the 2025 attack rate attributable to vaccination. Figure 5 shows these results, in which each column represents a different value of ξ. In the scenario in which recovered individuals are less susceptible to new infections (ξ = 0.5), vaccinating recovered individuals reduces the efficiency of the vaccination campaign, particularly at low vaccination coverage, as observed in the previous scenario (Figure 3). However, if the susceptibility of recovered individuals is higher than that of non-recovered individuals (ξ = 1.5), vaccinating recovered individuals leads to greater vaccination efficiency. As vaccine-induced population immunity increases, all scenarios converge to an approximate 80% reduction. When ξ = 1.0, no difference is observed between strategies targeting different proportions of recovered individuals. From top to bottom, the same pattern is observed across all three age groups.

## Discussion

In Botucatu, the number of cases in 2024 was nearly 10 times higher than the total recorded from 2014 to 2023, affecting at least 10% of the local population. Notably, the presence of Ae. aegypti mosquitoes in Botucatu was confirmed only between 2005 and 2010, based on oviposition trap data collected by local health authorities ^13^.

We used local incidence data to estimate the basic reproduction number of the disease. Our estimate, R_*0*_ = 1.57, is consistent with values reported by Marques et al. ^31^ during the 1990-1991 epidemic in 12 municipalities in the Ribeirão Preto area, São Paulo State, caused by the DENV-1 serotype, in which R_*0*_ ranged from 1.68 ± 0.12 to 2.49 ± 0.18. For other serotypes, Pinho et al. ^23^ reported a higher value of R_*0*_ = 2.85 during the circulation of DENV-2 in Salvador, Bahia State. In addition, Marques et al. ^31^ and Villela et al. ^24^ estimated R_*0*_ = 1.70 for DENV-3 during its initial appearance in Rio de Janeiro in 2002, and R_*0*_ = 1.25 for DENV-4 in 2014. It is important to emphasize that Equation 1 is not derived from the discrete model presented here. Instead, it originates from a previously developed continuous compartmental model that explicitly represents the mosquito population dynamics. It is used as a data-driven estimate of the basic reproduction number, providing an epidemiologically relevant measure of disease spread that enables comparison between the 2024 epidemic in Botucatu and previous outbreaks in the country. Other works have presented similar expressions based on the original article ^32^.

Moreover, it is not surprising that different values of R_*0*_ can be found. These differences arise from local variations in climatic conditions (e.g., temperature and humidity) and environmental factors (e.g., urban organization and waste disposal management) that modulate dengue transmission across various geographical regions.

In 2024, the Brazilian Ministry of Health prioritized municipalities for the Qdenga dengue vaccination campaign based on specific criteria: cities with more than 100,000 inhabitants, areas with high dengue transmission in 2023 and early 2024, and areas where DENV-2 was the predominant circulating serotype. This strategy aimed to maximize the campaign’s impact by focusing on densely populated and high-risk areas. A total of 521 Brazilian municipalities across 16 states and the Federal District were selected, with vaccination targeted at children aged 10 to 14 years - the group with the highest hospitalization rates ^33^.

The proposed model successfully captured the age-stratified dynamics and reproduced the epidemic peak observed in 2024. Notably, the parameter δ, representing the phase of the seasonal function, was estimated at approximately 0.48, peaking in the 10th Epidemiological Week - coinciding with the incidence point used to estimate R_*0*_ . Moreover, the model estimated the highest number of mosquito bites in the 10 to 14-year-old age group, consistent with findings on age-dependent exposure ^34^. This may help explain the elevated hospitalization rates observed in this age group and supports the prioritization strategy adopted by public health authorities. It is important to highlight that we do not claim that these estimations can be extended to other regions or municipalities, as they may strongly depend on the relationship between the number of human individuals and mosquitoes in the area, as well as other environmental factors ^35^. Instead, our estimations provide an adequate fit for our purposes, which is to study and analyze vaccination strategies and their efficiency in Botucatu.

Using the model, we explored hypothetical scenarios for 2025, considering the introduction of either a homologous or a heterologous dengue serotype, as well as different vaccination strategies depending on vaccine availability (Figures 3 and 5). It is important to note that, although vaccine effectiveness varies across serotypes, we varied the level of population immunity in the simulations, encompassing a wide range of vaccination coverage and vaccine effectiveness. If the same strain (DENV-1) were reintroduced in 2025 under the same conditions as in 2024, a significant reduction in the epidemic peak would be expected; however, an outbreak would still occur. In this scenario, vaccination plays a crucial role in reducing incidence, with an approximately 80% reduction achieved under full coverage of the 10-14 years old population. Notably, a similar level of reduction is maintained even in the case of heterologous serotype introduction. This is a key result that reinforces the importance of vaccination as a tool for epidemic control, particularly considering that the dengue vaccination campaign in Botucatu began in June 2025 with the Qdenga vaccine.

At lower levels of vaccination coverage, if a homologous serotype is introduced, vaccinating individuals who have already recovered is less effective, as these individuals are already immune to the circulating strain, thus leading to suboptimal use of available doses (Figure 3). Conversely, if a heterologous serotype is introduced, prior immunity may have a limited impact due to the typically short duration of cross-immunity, which is unlikely to persist beyond six months ^36^. In such cases, the optimal vaccination strategy depends on the immunological relationship between the previously and newly circulating serotypes. Some studies have estimated this influence, represented by ξ, showing that it may be greater or less than one depending on the serotype sequence ^37^. If prior infection increases susceptibility after the cross-immunity period (e.g., via antibody-dependent enhancement), vaccinating recovered individuals could improve campaign efficiency by reducing infections. Conversely, if prior infection induces residual protective immunity beyond the cross-immunity window, targeting non-recovered individuals would yield better results in terms of infection reduction (Figure 5).

Nevertheless, as vaccination coverage increases, particularly beyond 50%, the influence of dose allocation (whether to vaccinating recovered or non-recovered individuals) on overall campaign efficiency diminishes. All scenarios tend to converge toward similar levels of incidence reduction. This finding is especially relevant for public health planning, as serological testing to distinguish recovered from susceptible individuals, or to identify circulating serotypes, may be logistically complex and financially burdensome. Therefore, efforts should focus on maximizing vaccine coverage (above 50%) in the target population rather than implementing costly pre-vaccination screening procedures.

The number of dengue cases observed in Botucatu had a marked reduction in 2025 compared to 2024. Analyzing data up to the 39th Epidemiological Week, 16,214 notifications were registered in 2024 versus only 1,457 in 2025. While the results (Figures 2 and 4) indicate that population immunity may have contributed to a reduction in infections in the subsequent year, the mechanisms driving this reduction are multifactorial. For example, the onset of the La Niña phenomenon in 2025, contrasting with the El Niño conditions of the previous year, may have contributed to a lower larval index in Botucatu (according to the local health department, the percentage of buildings with larvae decreased from 6.3% to 3.5% in December 2024, and from 7% to 3% in January 2025).

Like any model, ours has limitations. For example, it does not account for individual heterogeneity or spatial structure - such as geographic barriers and social grouping - in which interactions are influenced by factors such as religion, social class, and other sociodemographic characteristics. However, given that dengue transmission occurs through a vector (mosquito), these limitations may be mitigated even in a homogeneous model such as the one presented here. Moreover, we assume that the Qdenga vaccine follows an all-or-nothing mechanism, i.e., it provides complete protection to a subset of vaccinated individuals. However, the literature classifies Qdenga as a leaky vaccine, that is, vaccinated individuals retain a probability of infection at every exposure event ^38^. As vaccination may lead to behavioral changes - reducing contact-avoidance behavior and potentially increasing exposure among vaccinated individuals - under a leaky-vaccine scenario, these individuals may face a higher risk of infection, which may impact vaccination efficiency ^39^. Nevertheless, because we conducted a comprehensive sensitivity analysis varying both the number of immune individuals - resulting from combinations of vaccination coverage and vaccine efficacy - and the proportion of recovered individuals who were vaccinated, we expect that the main effects of a leaky-vaccine dynamic are implicitly captured. Assuming that Qdenga provides 61% protection, similar to its reported efficacy against symptoms ^40^, and considering it as a leaky vaccine with protection reduced by up to 18%, the main conclusions regarding dose allocation and pre-vaccination screening procedures remain valid.

In summary, the model provided a good fit to the observed data and enabled the simulation of counterfactual scenarios to assess the potential impact of dengue vaccination strategies under conditions of limited vaccine supply. The optimal vaccination strategy depends on both the sequentially circulating serotype and the immunity profile of the population resulting from primary infection. As vaccine coverage increases, reductions in the attack rate converge toward a threshold level, regardless of the epidemiological status of vaccinated individuals.

## Data Availability

The sources of information used in the study are indicated in the body of the article. The codes are available in the repository: https://github.com/profthomasvilches/dengue_model_2025/tree/age_stratified.
